# 3,3′-Diindolylmethane Supplementation Maintains Oocyte Quality by Reducing Oxidative Stress and CEP-1/p53-Mediated Regulation of Germ Cells in a Reproductively Aged *Caenorhabditis elegans* Model

**DOI:** 10.3390/antiox11050950

**Published:** 2022-05-11

**Authors:** Mijin Lee, Esther Youn, Kyungsu Kang, Yhong-Hee Shim

**Affiliations:** 1 Department of Bioscience and Biotechnology, Konkuk University, Seoul 05029, Korea; miranda12@konkuk.ac.kr (M.L.); dptmej@konkuk.ac.kr (E.Y.); 2 Natural Product Informatics Research Center, Korea Institute of Science and Technology, Gangneung 25451, Gangwon-do, Korea; kskang@kist.re.kr

**Keywords:** 3,3′-diindolylmethane, antioxidant, reproductive aging, oocyte quality, CEP-1/p53, germ cell apoptosis, germ cell proliferation, mitochondrial function, oxidative stress, *Caenorhabditis elegans*

## Abstract

In recent decades, maternal age at first birth has increased, as has the risk of infertility due to rapidly declining oocyte quality with age. Therefore, an understanding of female reproductive aging and the development of potential modulators to control oocyte quality are required. In this study, we investigated the effects of 3,3′-diindolylmethane (DIM), a natural metabolite of indole-3-cabinol found in cruciferous vegetables, on fertility in a *Caenorhabditis elegans* model. *C. elegans* fed DIM showed decreased mitochondrial dysfunction, oxidative stress, and chromosomal aberrations in aged oocytes, and thus reduced embryonic lethality, suggesting that DIM, a dietary natural antioxidant, improves oocyte quality. Furthermore, DIM supplementation maintained germ cell apoptosis (GCA) and germ cell proliferation (GCP) in a CEP-1/p53-dependent manner in a reproductively aged *C. elegans* germ line. DIM-induced GCA was mediated by the CEP-1-EGL-1 pathway without HUS-1 activation, suggesting that DIM-induced GCA is different from DNA damage-induced GCA in the *C. elegans* germ line. Taken together, we propose that DIM supplementation delays the onset of reproductive aging by maintaining the levels of GCP and GCA and oocyte quality in a reproductively aged *C. elegans*.

## 1. Introduction

Reproductive aging is an emerging issue because the age at pregnancy has increased in modern society. The diminished ovarian reserve manifests as women age, and the remaining oocytes lose their reproductive potential with a high frequency of errors in DNA repair systems [1]. Therefore, advanced maternal age is markedly linked to various reproductive disorders, including infertility, miscarriage, and birth defects [2].

Oxidative stress is a major contributor to oocyte aging [3]. Aged oocytes are vulnerable to oxidative stress because of the reduced expression of antioxidant genes [4]. During reproductive aging, the accumulation of reactive oxygen species (ROS) can damage oocytes, resulting in infertility [3]. In aged oocytes, mitochondrial dysfunction is associated with the loss of mitochondrial membrane potential (MMP), reduced ATP production, and increased ROS production through defects in the electron transport chain [5]. Impaired mitochondria lead to chromosomal abnormalities, including telomere shortening, telomere loss, chromosome breakage, and chromosomal misalignment in aged oocytes, which are primarily observed in the oocytes of reproductively aged animals, from invertebrates to humans [2,5,6]. These findings suggest that infertility with advanced maternal age is caused by a decline in oocyte quality and increased oxidative stress. Many studies have shown that antioxidants contribute to enhancing oocyte quality and reproductive capacity in aged animals [7,8]. Among them, 3,3′-diindolylmethane (DIM) (Figure S1), the final metabolized product derived from indole-3-cabinol found in cruciferous vegetables such as cabbage and broccoli, has been extensively studied [9]. DIM shows beneficial effects against carcinogenesis [10,11,12] and resistance to heat shock and oxidative stress by regulating the level of ROS in yeast cells [13]. In *Caenorhabditis elegans*, DIM supplementation suppresses adipogenesis and improves intestinal permeability dysfunction following bacterial infection [14,15]. However, the effects of DIM supplementation on reproductive capacity with age remain largely unknown.

Recently, a systematic evaluation of the dietary potential of natural products to overcome clinical problems has emerged. The free-living nematode *C. elegans*, a simple animal model organism, has been extensively used to circumvent the limitations of human studies. *C. elegans* is a metazoan and has been widely used in laboratory conditions since being developed as an animal model by Sydney Brenner [16]. It has several advantages for the study of reproductive aging because of its simplicity and similar oogenesis process to that of mammals [17,18]. It is arrested at the meiotic cell cycle before fertilization, and chromosomal abnormalities are frequently observed in the aged oocytes of both *C. elegans* and humans with a decline in reproductive capacity [18]. In addition, it contains sequentially mitotic germ cells to meiotic germ cells, oocytes, and sperm in one gonad arm [19]. Therefore, observation of the reproductive aging progress from germ cell proliferation (GCP) to germ cell differentiation is feasible in a *C. elegans* model [20]. At the reproductively young adult stage, oocyte quality appears to be controlled by bidirectional crosstalk between germ cell apoptosis (GCA) and GCP in *C. elegans* hermaphrodites [21]. Reproduction then begins to decline as early as 3 days post the young adult stage with the reduction in GCP, and GCA transiently increased and then decreased during the reproductive process in *C. elegans* hermaphrodites [20,21]. Consequently, the balance between the GCA and GCP seems to be important for successful fertility and maintaining a healthy gonad.

In this study, we investigated whether DIM modulates oocyte quality during reproductive aging in *C. elegans*. We examined mitochondrial function in reproductively aged *C. elegans* to determine whether the oxidative stress accumulated during aging was suppressed by DIM supplementation. We also examined whether DIM supplementation delayed the onset of reproductive aging by analyzing the oocyte quality, GCA, and GCP.

## 2. Materials and Methods

### 2.1. C. elegans Strains

*C. elegans* strains were maintained at 15 °C on nematode growth medium (NGM) agar plates seeded with *Escherichia coli* strain OP50, as previously described [16]. The following strains were used in this study: N2 (*C. elegans* wild isolate, Bristol variety), VC289: *prdx-2(gk169)* II, SJ4100: *zcIs13[hsp-6p::GFP+ lin-15(+)]*, SJ4103: *zcIs14 [myo-3::GFP(mit)]*, WS1433: *hus-1(op241)* I; *unc-119(ed3)* III; *opIs34 [hus-1p::hus-1::GFP + unc-119(+)]*; MT1743: *ced-3(n718)* IV, TG12: *cep-1(lg12501)* I; *unc-119(ed4)* III; *gtIs1 [CEP-1::GFP + unc-119(+)]*, TJ1: *cep-1(gk138)* I, MT1082: *egl-1(n487)* V, FX536: *ced-13(tm536)* X, MT2551: *ced-4(n1162) dpy-17(e164)* III, DCL569: *mkcSi13 [sun-1p::rde-1::sun-1 3′UTR + unc-119(+)]* II; *rde-1(mkc36)* V; LD1: *ldIs7 [skn-1b/c::GFP + rol-6(su1006)]*.

### 2.2. DIM Supplementation

To examine the effect of DIM (Sigma-Aldrich, St. Louis, MO, USA) supplementation, each concentration (0, 10, 25, 50, 100 µM, and 1 M) dissolved in dimethyl sulfoxide (DMSO; Sigma-Aldrich, St. Louis, MO, USA) was diluted with *E. coli* OP50 solution. Cultured *E. coli* OP50 cells containing either DMSO or DIM were seeded on an NGM agar plate. Synchronized 24 or 48 h post L4-stage worms (day 2 adults, considered as young adults) and synchronized 96 h post L4-stage worms (day 4 adults, considered as aged adults) were examined. For instance, DIM supplementation to the aged worms (day 4 adults) was as follows: synchronized 72 h post L4-staged worms were supplemented with 100 µM DIM and examined after 24 h incubation at 20 °C in most of the experiments unless otherwise specified.

### 2.3. Mitochondrial ROS Assay

To measure the level of mitochondrial ROS production in the whole body or oocytes after DIM supplementation, CellROX^®^ Green (Invitrogen, Carlsbad, CA, USA) staining was performed, as previously described [22]. Briefly, the worms were stained with 5 µM CellROX^®^ Green for 20 min at 20 °C in the dark for the whole body. To investigate the level of mitochondrial ROS in the oocytes, worms were dissected in 10 μL of 5 μM CellROX^®^ Green solution on a glass slide and then incubated in a water chamber for more than 30 min at 20 °C. The mitochondrial ROS signal was captured under the same exposure conditions using a fluorescent microscope (Zeiss Axioskop, Oberkochen, Germany) and quantified using ImageJ software (National Institutes of Health, Bethesda, MD, USA ).

### 2.4. Mitochondrial Activity and MMP Assays

Mitochondrial activity and MMP in the whole body or oocytes were measured by MitoTracker Red and tetramethylrhodamine methyl ester (TMRM) staining, as previously described [22,23]. The worms were incubated for 3 h at 20 °C with 10 μM MitoTracker Red (Invitrogen, Carlsbad, CA, USA) and the *C. elegans* gonads were then dissected in 10 μL of 0.1% tetramisole on a slide glass to release oocytes. The staining signals were captured under the same exposure conditions using a fluorescent microscope (Zeiss Axioscope, Oberkochen, Germany) and quantified using ImageJ software. TMRM (Thermo Fisher Scientific, Waltham, MA, USA) staining was performed as previously described [22,24]. TMRM agar plates and NGM agar, containing a final concentration of 30 μM TMRM, were dried overnight and seeded with dead *E. coli* OP50 containing DMSO or DIM in the dark. The worms were then transferred to TMRM agar plates and incubated for 6 h at 20 °C, and whole body or oocytes released by dissection were observed with a fluorescent microscope (Zeiss Axioscope, Oberkochen, Germany).

### 2.5. Fluorescence Transgenic Worms Imaging Assay

To investigate the mitochondrial morphology with DIM supplementation, the transgenic strain SJ4103 was observed. SJ4103 contains the transgene *myo-3*::GFP(mit), which expresses mitochondrial GFP in the muscle tissue [25]. To investigate the mitochondrial stress response following DIM supplementation, the transgenic strain SJ4100 was used. SJ4100 contains an *hsp-6p*::GFP transgene that is driven by the *hsp-6* promoter and responds to mitochondrial protein misfolding [26]. The transgenic worms were mounted in 10 μL of 0.1% tetramisole on a slide glass. Live worm images were captured under a fluorescence microscope (Zeiss Axioscope, Oberkochen, Germany) and quantified using ImageJ software.

### 2.6. Embryonic Lethality, Unfertilized Oocytes, and Brood size Assays

To investigate the effect of DIM on reproductive capacity, worms were grown at 20 °C on an NGM agar plate and transferred to DMSO or DIM plates at 72 h post L4-stage for 24 h. Embryonic lethality was quantified as the percentage of non-hatched embryos out of the total number of embryos produced by day 4 adult worms supplemented with DMSO or DIM for 24 h at 20 °C. Unfertilized oocytes were distinguished as soft brown opaque round shapes compared with normal embryos. Brood size was quantified as the total number of progenies produced by an adult worm over six days.

### 2.7. Germline Dissection and Immunostaining Assay

The *C. elegans* gonads were dissected in 10 μL of 0.1% tetramisole on a slide glass and fixed by freeze cracking with liquid nitrogen and methanol–acetone fixation. Gonads were stained with primary and secondary antibodies. The specimens were counterstained in Vectashield with DAPI (Vector Laboratories, Burlingame, CA, USA) to detect DNA and were observed under a fluorescence microscope (Zeiss Axioscope, Oberkochen, Germany). The primary antibodies used were rabbit anti-GFP (1:500; Novus, Centennial, CO, USA) and rabbit anti-pH3 (1:500; Upstate Biotechnology, NY, USA). The secondary antibody used was anti-rabbit IgG (Alexa Fluor 488 conjugated; 1:500; Invitrogen, Carlsbad, CA, USA).

### 2.8. DNA Staining in Oocytes

To observe the oocyte chromosomes, the extracted gonads were fixed by freeze cracking with liquid nitrogen and methanol–acetone and were then stained in Vectashield with DAPI (Vector Laboratories, Burlingame, CA, USA). The chromosomal morphology of the oocytes was observed under a fluorescence microscope (Zeiss Axioscope, Oberkochen, Germany), according to a previously described method [22].

### 2.9. GCA Assay

To examine the effect of DIM supplementation on GCA, acridine orange (AO; Molecular Probes, Eugene, OR, USA) vital staining was performed after DIM supplementation. The worms were incubated for 90 min at 20 °C with 10 μg/mL AO in M9 buffer in the dark and then washed on new non-food plates. The number of AO-positive germ cells per gonad arm was counted using a fluorescence microscope (Zeiss Axioscope; Oberkochen, Germany).

### 2.10. RNA Interference (RNAi) Assay

For the RNAi of *cep-1*, feeding RNAi cultures were grown overnight in LB medium with 100 µg/mL ampicillin and incubated for 2–3 days at 20 °C to induce double-stranded RNA production on an NGM agar plate containing 0.2% lactose. The synchronized L4-staged worms were transferred to a feeding RNAi plate with the *E. coli* strain HT115 (DE3), transcribing double-stranded RNA (dsRNA) from L4440-derived vectors in which a *C. elegans* genomic F52B5.4 (*cep-1*) was inserted, for 24 h at 20 °C, as previously described [27]

### 2.11. Western Blot Assay

A total of 300 synchronized wild-type N2 worms at day 1 and day 4 adult stages supplemented with DMSO or DIM for 24 h were harvested in 10 μL of protease inhibitor cocktail per experiment and lysed at 100 °C for 10 min. Each sample (18 μL) was loaded onto a 4–12% SDS-PAGE gel (Thermo Fisher Scientific, Waltham, MA, USA) and transferred to nitrocellulose membrane (PROTRAN BA83, Whatman, Sigma-Aldrich, St. Louis, MO, USA), and the procedure was followed as previously described [22]. Primary antibodies used were rabbit anti-CED-9 (1:1000; Santa Cruz Biotechnology, Dallas, TX, USA) and mouse anti-α-tubulin (1:1000; Sigma-Aldrich, St. Louis, MO, USA). The secondary antibodies used were horseradish peroxidase (HRP)-conjugated goat anti-rabbit IgG (1:1000; Santa Cruz Biotechnology, Dallas, TX, USA) and HRP-conjugated donkey anti-mouse IgG (1:1000; Jackson ImmunoResearch, West Grove, PA, USA). Blots were visualized with an ECL western blot detection kit (Amersham, GE Healthcare Life Sciences, Pittsburgh, PA, USA), detected using the LAS-3000 image analyzer, and quantified using ImageJ software.

### 2.12. Quantification of Fluorescence Image Using ImageJ Software

For fluorescence intensity quantification, ImageJ software (NIH, http://imagej.nih.gov/ij, accessed on 15 February 2022) was used, as previously described [28]. Fluorescence images were captured using a Zeiss Axioscope 2 plus fluorescence phase-contrast microscope with an HBO 100 fluorescent illuminator (Zeiss Axioscope, Oberkochen, Germany) connected to a Hamamatsu ORCA-ER digital camera (Hamamatsu, Shizuoka, Japan). The region of interest (ROI) with the fluorescence was drawn and then the mean pixel intensities of fluorescence area per image were calculated using the ‘measurement’ tool in ImageJ. The average measured value of each fluorescence of the worm is represented as a bar graph with dots in the figures.

### 2.13. Statistical Analysis

Statistical analysis was performed using the Prism software package (version 7; GraphPad Software; https://www.graphpad.com, accessed on 11 February 2022) to calculate the *p*-value using a one-way ANOVA with Tukey’s post-hoc test, multiple *t*-tests, and two-way ANOVA with Tukey’s post-hoc test or Sidak’s multiple comparisons test, chi-square test. Differences were considered statistically significant at *p* < 0.05. T-bars represent mean ± standard deviation (SD). All experiments were repeated at least thrice.

## 3. Results

### 3.1. DIM Supplementation Improves Mitochondrial Function and Morphology in C. elegans

**Figure 1:**
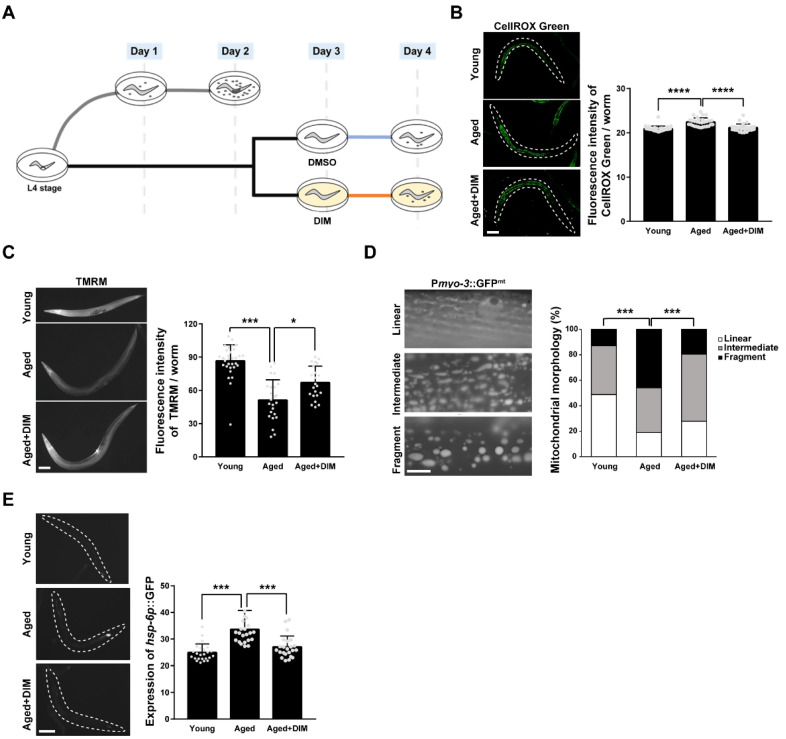
3,3′-Diindolylmethane (DIM) supplementation improved mitochondrial function in *Caenorhabditis elegans*: (**A**) The experimental scheme for the effect of DIM supplementation on reproductive capacity. (**B**) Representative images and a quantification graph of mitochondrial reactive oxygen species (mtROS) levels using CellROX Green staining were shown in wild-type N2 at the day 1 adult stage (Young, *n* = 30) and wild-type N2 supplemented with DMSO (Aged, *n* = 30) or DIM (Aged+DIM, *n* = 30) at the day 3 adult stage for 24 h. Error bars represent SD. ****, *p* < 0.0001 (one-way ANOVA with Tukey’s post-hoc test). (**C**) Representative images and a quantification graph of mitochondrial membrane potential levels using tetramethylrhodamine methyl ester (TMRM) staining were shown in wild-type N2 at the day 1 (Young, *n* = 31) and day 4 adult stages supplemented with DMSO (Aged, *n* = 24) or DIM (Aged+DIM, *n* = 21) for 24 h. Error bars represent SD. *, *p* < 0.05, ***, *p* < 0.001 (one-way ANOVA with Tukey’s post-hoc test). (**D**) Representative images and a percentage graph indicate mitochondrial morphology in the muscle using P*myo-3*::GFP^mt^ transgenic animals at the day 1 (Young, *n* = 39) and day 4 adult stages supplemented with DMSO (Aged, *n* = 37) or DIM (Aged+DIM, *n* = 36) for 24 h. The analysis of mitochondrial morphology was classified into three categories: (1) linear, (2) intermediate, and (3) fragmented. Scale bars represent 10 μm. ***, *p* < 0.001 (chi-square test). (**E**) Representative images and quantification graph indicate the levels of expression of *hsp-6p::GFP* transgenic animals at the day 1 (Young, *n* = 24) and day 4 adult stages supplemented with DMSO (Aged, *n* = 22) or DIM (Aged+DIM, *n* = 23) for 24 h. Error bars represent SD. ***, *p* < 0.001 (one-way ANOVA with Tukey’s post-hoc test).

In self-fertile *C. elegans* hermaphrodites, reproduction is active from the day 1 adult stage (24 h post L4 stage) when fertilization begins, is maximized between the day 3 and day 4 adult stages, and then starts to decline when fewer fertilized embryos are produced [20]. To investigate whether DIM supplementation suppresses oxidative stress during reproductive aging as a dietary antioxidant, we measured the levels of ROS in young and aged adult *C. elegans.* As shown in the experimental scheme, we designated day 1 and day 2 adults as reproductively young and day 4 adults as reproductively aged worms in this study (Figure 1A). The day 4 adult worms were obtained by incubating day 3 adults in NGM plates containing DMSO (blue line, aged) or DIM (orange line, aged + DIM) for 24 h before observation (Figure 1A). Previously, we reported that DIM supplementation at 100 μM improved intestinal permeability and extended the lifespan of *C. elegans* infected with pathogenic bacteria [15]. Thus, we first examined whether the level of mitochondrial ROS (mtROS) can be modulated by 100 μM DIM supplementation. To detect mtROS levels, we stained the worms with CellROX Green dye, which binds to oxidatively damaged mitochondrial DNA [22,29], in day 1 adults (young) and day 4 adults (aged). The level of mtROS was significantly increased in aged worms compared to young worms, but the increased level of mtROS in aged worms was indeed suppressed by DIM supplementation (Figure 1B). This finding was supported by the finding that DIM supplementation reduces mtROS levels in *prdx-2* mutants, which are defective in detoxifying hydrogen peroxide [30] (Figure S2). These results confirmed that DIM supplementation reduces free radicals as a natural antioxidant in *C. elegans*. Considering that a high level of mtROS production is caused by MMP disruption with aging, or vice versa [31], we measured MMP using TMRM staining, which is a fluorescent dye accumulated in healthy mitochondrial membranes [32]. MMP levels were significantly reduced in aged worms compared to those in young worms, and the reduced MMP levels in aged worms were recovered by DIM supplementation (Figure 1C).

As aging progresses, the fragmented mitochondrial morphology increases owing to excessive mitochondrial fission to remove damaged mitochondria [33]. Therefore, we examined mitochondrial morphology in the muscle of P*myo-3*::GFP^mt^ transgenic worms with or without DIM supplementation. The proportion of fragmented mitochondria in aged worms was approximately 46%, while it was 13% in young worms (Figure 1D). However, the proportion of fragmented mitochondria was reduced to 19% by DIM supplementation in aged worms, which was an almost similar level to that observed in young worms (Figure 1D), suggesting that DIM supplementation can suppress mitochondrial dysfunction during aging. As the mitochondrial unfolded protein response (mitoUPR) is induced by mitochondrial dysfunction [34], we further analyzed the level of mitoUPR by measuring the expression level of the *hsp-6p::GFP* transgene, a reporter for mitochondrial stress. The expression level of *hsp-6p*::GFP was increased in aged worms compared to that in young worms and was further suppressed by DIM supplementation in aged worms (Figure 1E), suggesting that DIM supplementation decreases mitochondrial stress. Taken together, DIM supplementation reduced oxidative stress and maintained mitochondrial function in the aged worms.

### 3.2. DIM Supplementation Improves Reproductive Capacity during Reproductive Aging

**Figure 2:**
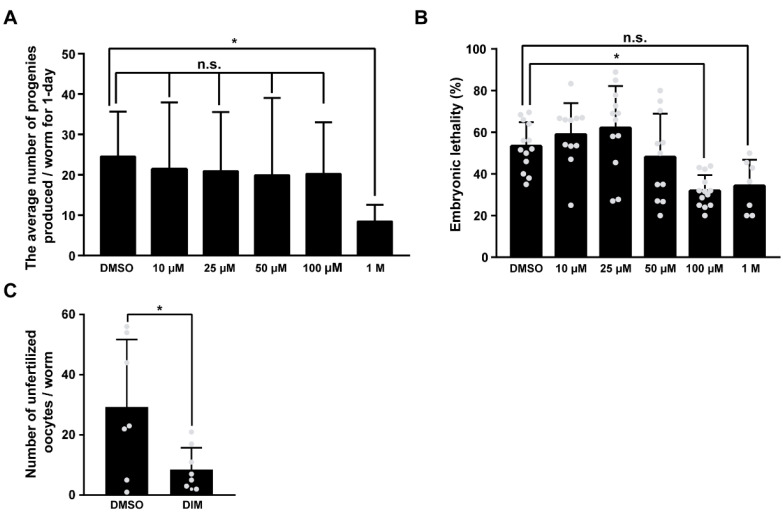
DIM supplementation improved the reproductive capacity in reproductively aged *Caenorhabditis elegans*: (**A**) The graph indicates the number of total embryos in wild-type N2 supplemented with DMSO or DIM (10, 25, 50, 100 μM, and 1 M concentrations) at the day 3 adult stage for 24 h. Error bars represent SD. n.s., not significant., *, *p* < 0.05 (one-way ANOVA with Turkey post-hoc test). Each condition, *n* > 8. (**B**) The graph indicates the percentages of unhatched embryos per total embryos in wild-type N2 supplemented with DMSO or DIM (10, 25, 50, 100 μM, and 1 M concentrations) at the day 3 adult stage for 24 h. Error bars represent SD. n.s., not significant., *, *p* < 0.05 (one-way ANOVA with Turkey post-hoc test). Each condition, *n* > 8. (**C**) The graph indicates the number of unfertilized oocytes produced from day 4 adult wild-type N2 worms supplemented with DMSO or DIM. Error bars represent SD. *, *p* < 0.05 (multiple *t*-tests).

The effect of antioxidants on oocyte quality in a variety of animal models has been studied related to mitochondrial function [35]. Energy metabolism and ROS production, which are two major indicators of mitochondrial function, contribute to the quality of germ cells and fertility in *C. elegans* [23]. Therefore, we examined whether DIM supplementation improved the reproductive capacity of *C. elegans.* To investigate the effect of DIM supplementation on reproductive capacity, we first determined the optimal DIM concentration for the study by measuring brood size and embryonic lethality in aged worms supplemented with 10, 25, 50, 100 μM, and 1 M DIM (Figure 2A,B). Except for the 1 M DIM supplementation, the other test concentrations showed a similar number of progeny (Figure 2A). Aged worms supplemented with 100 μM DIM showed the lowest number of dead embryos, while the number of progenies was maintained at the level of the control group without DIM supplementation, suggesting that the most abundant progeny was alive with 100 μM DIM supplementation (Figure 2B). These findings imply that the effects of DIM supplementation on fertility are dose-dependent. Therefore, 100 μM of DIM was used in this study.

Because embryonic lethality is closely linked to oocyte quality in *C. elegans* [22,36], low embryonic lethality with DIM supplementation suggests increased oocyte quality. Therefore, we first counted the number of unfertilized oocytes produced each day with DMSO or DIM supplementation from day 1 to day 6, along with the number of hatched and unhatched embryos (Figure S3). Interestingly, the number of unfertilized oocytes and unhatched dead embryos was significantly reduced from day 4 of DIM supplementation (Figure 2C and Figure S3). These findings indicate that DIM supplementation has a strong beneficial effect on the reproductive capacity of reproductively aged worms. Taken together, these results suggest that DIM supplementation improves the oocyte quality and reproductive capacity in aged worms.

### 3.3. DIM Supplementation Improves the Mitochondrial Function in Aged Oocytes

**Figure 3:**
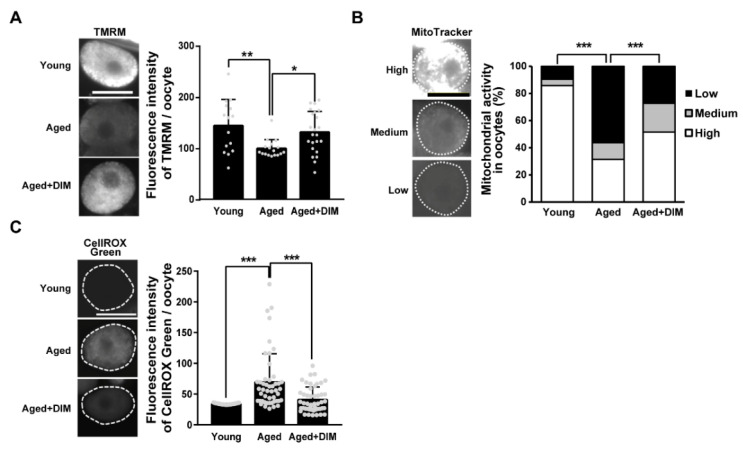
DIM supplementation improved the mitochondrial function in the aged *Caenorhabditis elegans* oocytes: (**A**) Representative images and quantification graph of mitochondrial membrane potential levels using TMRM staining were shown in the oocytes produced by day 2 adults (Young, *n* = 15) and day 4 adults supplemented with DMSO (Aged, *n* = 17) or DIM (Aged+DIM, *n* = 23). Error bars represent SD. *, *p* < 0.05, **, *p* < 0.01 (one-way ANOVA with Tukey’s post-hoc test). (**B**) Representative images and a percentage graph of mitochondrial activity using MitoTracker staining were shown in the oocytes produced by day 2 adults (Young, *n* = 42) and day 4 adults supplemented with DMSO (Aged, *n* = 105) or DIM (Aged+DIM, *n* = 95). The analysis of mitochondrial activity was classified into three categories: (1) high, (2) medium, and (3) low using the fluorescence intensity of MitoTracker dye. ***, *p* < 0.001 (chi-square test). (**C**) Representative images and quantification graph of mitochondrial ROS levels using CellROX Green staining were shown in the oocytes produced by day 2 adults (Young, *n* = 37) and day 4 adults supplemented with DMSO (Aged, *n* = 48) or DIM (Aged+DIM, *n* = 48). Error bars represent SD. ***, *p* < 0.001 (one-way ANOVA with Tukey’s post-hoc test). Scale bars represent 20 μm.

Normal mitochondrial function is essential for maintaining oocyte quality [5,22]. Accordingly, we previously reported a decreased level of MMP and increased mtROS production in reproductively aged *C. elegans* oocytes [22]. Therefore, we examined whether DIM supplementation improved mitochondrial activity in aged oocytes produced from day 4 adults. MMP levels were significantly lower in aged oocytes than in young oocytes produced from day 2 adults. However, DIM supplementation resulted in higher MMP levels in aged oocytes than in worms without DIM supplementation (Figure 3A). Next, we stained the worms with the MitoTracker dye as a fluorescent probe that accumulates in active mitochondria by passively diffusing across the plasma membrane. Depending on the intensity of the MitoTracker dye, we categorized the mitochondrial phenotype of the oocytes as low, medium, and high (Figure 3B). Approximately 56% of aged oocytes showed a low mitochondrial activity, while it was 9.5% in young oocytes. Fewer oocytes with DIM supplementation (Aged+DIM) showed lower mitochondrial activity than aged oocytes without DIM supplementation (Aged) (Figure 3B). Because mitochondrial dysfunction is highly associated with mitochondrial oxidative stress [5], we also measured mtROS production in oocytes. Aged oocytes showed an increased level of mtROS compared to young oocytes (Figure 3C). However, DIM supplementation suppressed mtROS levels in aged oocytes to levels similar to those observed in young oocytes (Figure 3C). Taken together, these results suggest that DIM supplementation enhances mitochondrial function in aged oocytes, similar to the effects on oocyte quality.

### 3.4. DIM Supplementation Decreased Chromosomal Abnormality in Aged Oocytes

**Figure 4:**
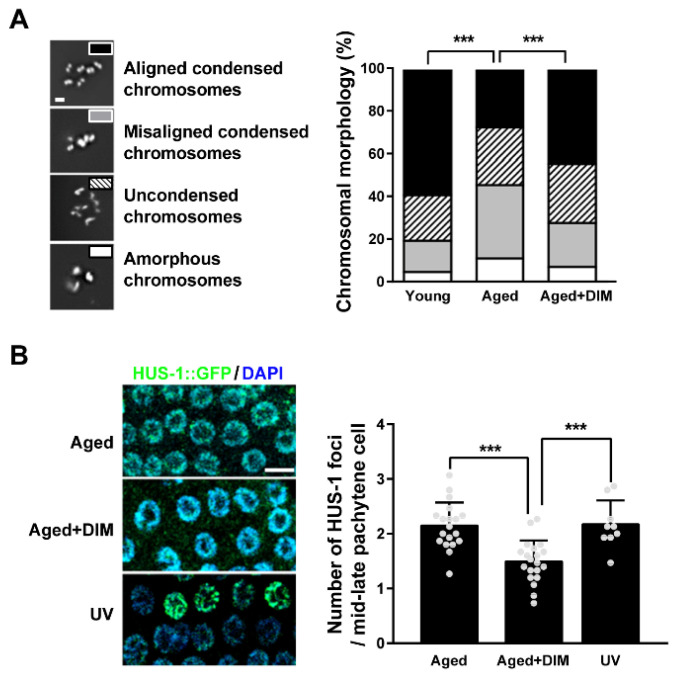
DIM supplementation decreased chromosomal abnormality in reproductively aged *Caenorhabditis elegans*: (**A**) Representative images and a percentage graph of chromosomal aberrations were shown using DNA staining in the oocytes produced by day 2 adults (Young, *n* = 105) and day 4 adults supplemented with DMSO (Aged, *n* = 89) or DIM (Aged+DIM, *n* = 115). The analysis of chromosomal aberrations was classified into four categories: (1) aligned condensed chromosomes, (2) misaligned condensed chromosomes, (3) uncondensed chromosomes, and (4) amorphous chromosomes. Scale bars represent 2 μm. ***, *p* < 0.001 (chi-square test). (**B**) Representative images of the HUS-1 foci (green) in the mid-late pachytene germ cells were shown using anti-GFP immunostaining. The graph indicated the numbers of HUS-1 foci per germ nucleus in day 4 adults supplemented with DMSO (Aged, *n* = 20) or DIM (Aged+DIM, *n* = 20) for 24 h. UV treatment was used for the positive control (UV, *n* = 9). Error bars represent SD. ***, *p* < 0.001 (one-way ANOVA with Tukey’s post-hoc test). Scale bars represent 10 μm.

In aged oocytes, the high frequency of errors in chromosomal segregation causes infertility in *C. elegans* and mammals [5,36,37]. To examine whether DIM supplementation can maintain chromosomal integrity in aged oocytes, we examined chromosomes in aged oocytes by DNA staining. The major feature of mature *C. elegans* oocytes is the presence of six pairs of aligned and condensed chromosomes in the nuclei of young oocytes [38]. Mature oocytes are at the diakinesis stage in meiotic prophase I during oogenesis before fertilization [39]. Chromosomal abnormalities in the oocytes were classified into four classes: aligned and condensed (normal six-bivalent phenotype), misaligned and condensed, uncondensed, and amorphous (Figure 4A). Approximately 59% of oocytes contained aligned and condensed chromosomes (six-bivalents) with normal DNA morphology in young oocytes, while it was 27% in aged oocytes. However, DIM supplementation showed 44% of aligned and condensed chromosomes in aged oocytes (Figure 4A). These findings indicate that DIM supplementation maintains the chromosomal integrity of aged oocytes.

Chromosomal missegregation in oocytes can be induced by DNA damage in the germ line of *C. elegans* [40]. During reproductive aging, damaged DNA accumulates in the *C. elegans* germ line through an inefficient DNA repair system, which eventually causes chromosomal aberrations [41]. To verify whether the alleviation of chromosomal aberrations by DIM supplementation was associated with reduced DNA damage, the number of HUS-1 foci per germ cell was counted in *hus-1*::GFP transgenic worms. The foci of HUS-1 form in chromatin when spontaneous mutations, chromosome nondisjunction, and telomere shortening occur due to DNA damage [42]. The number of HUS-1 foci increased in the aged germ line, as in worms exposed to UV light (Figure 4B). Interestingly, the increased number of HUS-1 foci in the aged germ line was suppressed by DIM supplementation (Figure 4B). These results suggest that DIM supplementation decreases chromosomal abnormalities in aged oocytes by reducing DNA damage in the germ line of *C. elegans.*

### 3.5. DIM Supplementation Increased GCA and GCP

**Figure 5:**
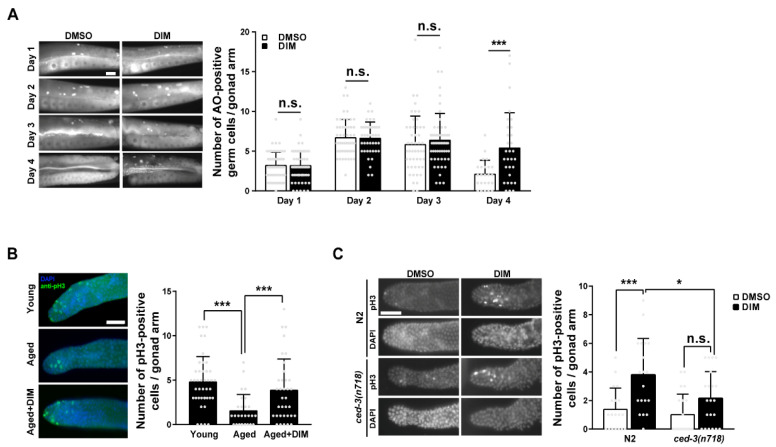
DIM supplementation increased germ cell apoptosis and germ cell proliferation in reproductively aged *Caenorhabditis elegans*: (**A**) Representative images and a quantification graph indicate average numbers of acridine orange (AO)-positive germ cells per gonad arm using AO staining in the wild-type N2 at each day after DMSO or DIM supplementation for 24 h. Each condition, *n* > 30. Error bars represent SD. n.s., not significant., ***, *p* < 0.001 (multiple *t*-test). (**B**) Representative images and quantification graph indicate average numbers of phosphohistone H3 (pH3)-positive germ cells per gonad arm using anti-pH3 immunostaining in day 2 adults (Young, *n* = 43) and day 4 adults supplemented with DMSO (Aged, *n* = 28) or DIM (Aged+DIM, *n* = 35). Error bars represent SD. ***, *p* < 0.001 (one-way ANOVA with Tukey’s post-hoc test). (**C**) Representative images and quantification graph indicate average numbers of phosphohistone H3 (pH3)-positive germ cells per gonad arm using anti-pH3 immunostaining in day 4 adult *ced-3(n718)* mutants supplemented with DMSO or DIM (each condition, *n* > 20). Error bars represent SD. n.s., not significant., *, *p* < 0.05, ***, *p* < 0.001 (two-way ANOVA with Tukey’s post-hoc test). Scale bars represent 20 μm.

GCA plays an essential role in maintaining the quality of oocytes by allowing the efficient allocation of resources to develop germ cells into oocytes in *C. elegans* [43]. Therefore, we examined the levels of GCA in reproductively aged worms supplemented with DIM. We analyzed GCA using AO staining in *C. elegans* during reproductive aging. AO intercalates into double-stranded nucleic acids in cells at an acidic pH. Therefore, AO-positive germ cells indicate apoptotic germ cells [44]. In a previous report, the highest number of AO-positive germ cells was observed in day 2 young adults, but it was significantly decreased in day 4 aged adults [20,22]. Surprisingly, the number of AO-positive germ cells remained high even in aged worms after DIM supplementation, whereas it severely decreased in aged worms without DIM supplementation (Figure 5A). This finding implies that DIM supplementation delays reproductive aging by maintaining the level of GCA, and the increased level of GCA is linked to improved oocyte quality.

The ratio between GCP and GCA is regulated to maintain gonad biomass in young adults [21]. Therefore, we examined whether DIM supplementation affects the level of GCP in reproductively aged worms by analyzing the proliferative germ cells in the distal gonad with anti-phosphohistone H3 (pH3) staining, which stains mitotic germ cells. As expected, the number of pH3-positive germ cells was significantly lower in aged worms than that in young worms (Figure 5B). However, the number of pH3-positive germ cells increased in the aged germ line with DIM supplementation (Figure 5B), suggesting that DIM supplementation also maintains GCP in aged worms, similar to GCA. Taken together, these findings support the possibility that DIM supplementation delays reproductive aging by maintaining GCA and GCP levels in aged germ lines, similar to those in young adults.

To determine whether GCA and GCP induced by DIM supplementation in reproductively aged worms were linked, we performed anti-pH3 staining in apoptosis-defective *ced-3* mutants at the aged adult stage. CED-3 is a homolog of mammalian caspases and is the final effector of apoptosis in *C. elegans* [45]. Interestingly, the number of pH3-positive germ cells was still increased in *ced-3* mutants with DIM supplementation compared to control worms without DIM, although the difference was not significant (Figure 5C). However, the number of pH3-positive germ cells was significantly decreased in *ced-3* mutants compared to that in control wild-type N2 worms with DIM supplementation (Figure 5C). These results suggest a possible link between the balance of proliferation and apoptosis in the aged germ line with DIM supplementation, but there is an intrinsic level of GCP that exists independent of GCA. Taken together, we propose that DIM supplementation contributes to the maintenance of germline quality by maintaining GCA and GCP levels in *C. elegans* during reproductive aging.

### 3.6. GCA and GCP Were Maintained with DIM Supplementation Depending on Germline CEP-1 Activity in Reproductively Aged Worms

**Figure 6:**
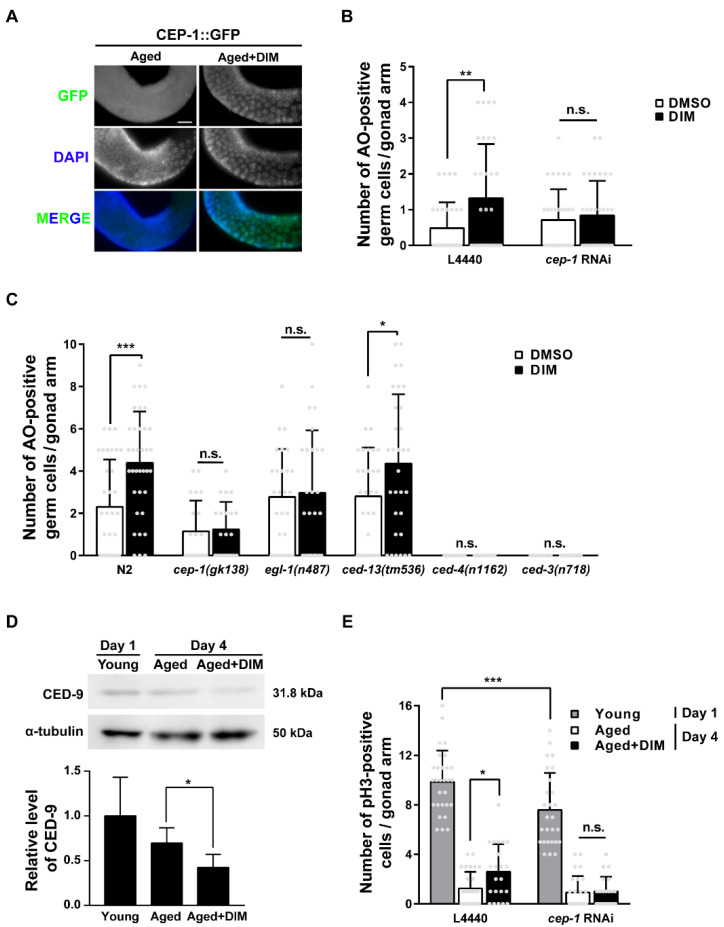
The germ cell apoptosis and germ cell proliferation induced by DIM supplementation were dependent on germline CEP-1 activity in reproductively aged *Caenorhabditis elegans*: (**A**) Representative images of the CEP-1 (green) in the late pachytene region were shown using anti-GFP immunostaining in CEP-1::GFP transgenic animals supplemented with DMSO (Aged, *n* = 28) or DIM (Aged+DIM, *n* = 27) at the day 3 adult stage for 24 h. (**B**) The graph indicates average numbers of AO-positive germ cells per gonad arm using AO staining in the knockdown of *cep-1* in the germ line (each condition, *n* = 31) of day 4 adults. The synchronized L4-staged DCL569 (germline-specific RNAi) strains were transferred to L4440 or *cep-1* feeding RNAi plate for 24 h, then transferred to the NGM plate. The day 3 adult worms were supplemented with DMSO or DIM for 24 h. Error bars represent SD. n.s., not significant., **, *p* < 0.01 (two-way ANOVA with Tukey’s post-hoc test). (**C**) The graph indicates average numbers of AO-positive germ cells per gonad arm using AO staining shown in the adult wild-type N2, *cep-1(gk138), egl-1(n487), and ced-13(tm536), ced-4(n1162), ced-3(n718)* mutants supplemented with DMSO or DIM at the day 3 adult stage for 24 h. (Each condition, *n* > 22). Error bars represent SD. n.s., not significant., *, *p* < 0.05, ***, *p* < 0.001 (two-way ANOVA with Sidak’s multiple comparisons test). (**D**) Representative images and a quantification graph indicate western blot analysis of CED-9 in young (*n* = 300), aged (*n* = 300), and aged+DIM (*n* = 300). The expression level of CED-9 was normalized to that of α-tubulin (loading control) on the same lane, and the relative level of CED-9 was converted to a relative value against that of Day 1 as 1. Error bars represent SD. *, *p* < 0.05 (two-way ANOVA with Tukey’s post-hoc test). (**E**) The graph indicates average numbers of phosphohistone H3 (pH3)-positive germ cells per gonad arm using anti-pH3 immunostaining in the knockdown of *cep-1* in the germ line supplemented with DMSO or DIM. Each condition, *n* > 21. The synchronized L4-staged DCL569 (germline-specific RNAi) strains were transferred to L4440 or *cep-1* feeding RNAi plate. Day 3 adults were supplemented with DMSO or DIM for 24 h. Error bars represent SD. n.s., not significant., *, *p* < 0.05, ***, *p* < 0.001 (two-way ANOVA with Tukey’s post-hoc test).

In *C. elegans*, the p53-like protein CEP-1 is essential for controlling the quality of germ cells through the regulation of GCA and GCP [46,47]. Therefore, we investigated whether DIM supplementation maintains reproductive health through CEP-1 activity by observing the expression pattern of CEP-1::GFP in the germ line of reproductively aged worms. We observed germ cells in the late pachytene region, where germ cells undergo apoptosis and CEP-1 expression is observed [48]. The expression of CEP-1::GFP was evident in the germ cells of the pachytene region in aged worms with DIM supplementation compared to the control group without DIM (Figure 6A). This finding indicates that DIM supplementation induced CEP-1 expression in the germ line and that the effects observed by DIM supplementation are possibly dependent on CEP-1 activity. To determine whether GCA induced by DIM supplementation was dependent on CEP-1 activity in aged worms, we performed germline-specific RNAi of *cep-1* using the DCL569 strain (Figure 6B). The DCL569 strain was constructed for exclusive RNAi knockdown in the germ line by expressing *rde-1* under the *sun-1* germline-specific promoter in the *rde-1* mutant [49]. Therefore, the function of *cep-1* in the germ line can be analyzed using *cep-1* RNAi in the DCL569 strain. Furthermore, *cep-1* RNAi was performed during the late L4 stage to avoid somatic developmental defects. While the number of AO-positive germ cells increased in aged worms with DIM supplementation with control RNAi, the level of GCA was not further increased by DIM supplementation in *cep-1* RNAi-depleted worms (Figure 6B). These results indicate that DIM supplementation induces CEP-1-mediated GCA, which was further confirmed by examining the downstream genes of CEP-1 in the apoptotic pathway.

In *C. elegans*, CEP-1 transcriptionally activates the pro-apoptotic BH3-only proteins EGL-1 and CED-13 [50]. These proteins bind to the anti-apoptotic Bcl-2 protein CED-9 to release the pro-apoptotic Apaf-1 protein, CED-4. CED-4 then activates the caspase protein CED-3 to induce GCA in *C. elegans* [50]. The number of apoptotic germ cells increased in wild-type N2 and *ced-13* mutants with DIM supplementation, whereas the number of apoptotic germ cells did not increase in *cep-1* and *egl-1* mutants, and no GCA was observed in *ced-4* and *ced-3* mutants, as expected (Figure 6C). These findings indicate that the GCA induced by DIM supplementation in reproductively aged worms is activated by the CEP-1-EGL-1 pathway. Additionally, we measured the mRNA levels of *egl-1* and *ced-13* in wild-type N2 adults supplemented with DIM (Figure S4). Remarkably, the mRNA levels of *egl-1* and *ced-13* were not increased in young worms supplemented with DIM compared to the control worms without DIM supplementation (Figure S4A,B). However, DIM supplementation significantly increased the mRNA level of *egl-1* in aged worms (Figure S4C). Unfortunately, the mRNA level of *ced-13* was not detectable (Figure S4D). Taken together, DIM supplementation induced GCA through the CEP-1-EGL-1 apoptotic pathway in the *C. elegans* germ line.

We further examined the protein levels of CED-9 by western blotting. This was decreased by DIM supplementation in aged worms (Figure 6D and Figure S5), indicating that the increased level of GCA by DIM supplementation was due to the reduced level of CED-9 in response to the CEP-1-EGL-1 pathway in reproductively aged worms. As shown in Figure 6D, CED-9 expression was higher in day 1 young worms than in day 4 aged worms, although GCA was normally active in day 1 adults compared to day 4 adults [20]. This may be due to the presence of embryos in the uterus of day 1 adults because CED-9 is also required for normal development and is highly expressed during embryogenesis to prevent apoptosis [51].

GCP is also regulated by the activation of CEP-1 in the absence of DNA damage [52]. Therefore, we further verified the possibility that DIM supplementation induces GCP through CEP-1 activity. The number of anti-pH3 positive mitotic germ cells was measured in worms with or without DIM supplementation after the germline-specific *cep-1* RNAi using the DCL569 strain by staining with anti-pH3 antibody. The number of anti-pH3 positive germ cells was decreased, but not null, in the distal gonad of young worms by *cep-1* RNAi compared to that of the control worms, indicating that germline *cep-1* is partially required for GCP (Figure 6E). However, the number of pH3-positive germ cells in the distal gonad of the aged worms with control RNAi increased with DIM supplementation compared to that without DIM supplementation (Figure 6E). However, DIM supplementation did not increase the number of pH3-positive germ cells in aged worms with germline-specific *cep-1* RNAi (Figure 6E), suggesting that the increased level of GCP induced by DIM supplementation in aged worms is dependent on germline CEP-1 activity. Taken together, our findings demonstrate that DIM supplementation can regulate reproductive capacity during reproductive aging through CEP-1-mediated GCA and GCP in the *C. elegans* germ line.

## 4. Discussion

The beneficial effects of DIM as an antioxidant have been studied in various model organisms including flies, mice, and mammalian cells [53,54,55,56]. Here, we investigated the effects of DIM supplementation on fertility using a reproductively aged *C. elegans* animal model. In the animal aging process, mitochondrial dysfunction gradually increases with high levels of ROS, resulting in aging-related diseases [57]. In the present study, we observed that the increased level of ROS in reproductively aged worms was reduced, and the mitochondrial function and morphology were improved by DIM supplementation. Therefore, DIM exerts potential anti-aging effects that maintain reproductive health through the improved mitochondrial function and the reduced oxidative stress in the *C. elegans* germ line.

In response to oxidative stress, the expression of antioxidant enzyme genes, including *prdx-2* (peroxiredoxins), is transcriptionally activated in *C. elegans* [30]. Strong expression of nuclear factor erythroid-2-related factor (Nrf2) was reported to be induced by DIM supplementation [58]. Considering that Nrf2 promotes the expression of genes encoding phase II drug-metabolizing enzymes and antioxidant enzymes by binding to cis-regulatory elements, called antioxidant response elements [59], and Nrf2 is a homolog of *C. elegans* skinhead-1 (*skn-1),* we examined the expression of *skn-1b/c*::GFP and nuclear localization of SKN-1 for its activity after DIM supplementation. SKN-1 is also required for the balance between mitochondrial biogenesis and mitophagy in *C. elegans* [60], suggesting that improved mitochondrial function is associated with SKN-1. However, SKN-1 was not activated by DIM supplementation, indicating that the beneficial effects of DIM on mitochondrial function were independent of SKN-1 activity (Figure S6). The putative targets of DIM supplementation in response to oxidative stress in reproductively aged *C. elegans* remain elusive.

Our data demonstrate that DIM maintains the level of GCA in aged worms. GCA is essential for maintaining the fertility of *C. elegans* hermaphrodites, particularly under stressful conditions, including oxidative stress [61]. GCA plays a key role in controlling the quality of oocytes, which decreases with age, and the reduced GCA levels are highly correlated with increased embryonic lethality [22,43]. These findings indicate that GCA indeed regulates oocyte quality. Notably, in this study, we showed that DIM supplementation activated CEP-1/p53, a pro-apoptotic regulator, in the germ line of aged worms. This result is consistent with a previous report in which an antioxidant, clove essential oil, was found to improve fertility through CEP-1-mediated GCA in *C. elegans* [62]. It has been reported that CEP-1 is mainly activated to promote DNA damage-induced GCA [46,63]. Contrary to that, in this study, DIM-induced GCA in reproductively aged worms was independent of DNA damage because we observed a lower number of HUS-1 foci with DIM supplementation than in those without DIM supplementation. HUS-1 is a conserved DNA damage response protein in the germ line of *C. elegans* [42]. These findings imply that DIM supplementation maintains the quality of germ cells in reproductively aged worms by increasing the level of CEP-1-dependent GCA without DNA damage. Considering that hyperproduced ROS directly causes DNA damage by oxidizing nucleoside bases during aging [64], DIM was supposed to contribute to reducing oxidative DNA damage. Consistently, DIM supplementation reduced oxidative DNA damage through Nrf2 signaling in a mouse model [65,66]. Based on these findings, we propose that DIM, as an antioxidant, may reduce oxidative DNA damage and activate CEP-1-mediated GCA independently of DNA damage.

Interestingly, we found that DIM supplementation increased the levels of both GCP and GCA, and that both were dependent on CEP-1 activity. It has been reported that CEP-1 is an essential regulator in controlling GCP and GCA [47,48]. With age, declining GCP and increasing or decreasing GCA are observed in *C. elegans*, resulting in germline dysfunction and infertility [20,43]. In addition, the amount of *cep-1* transcript is reduced with age [67], and age-related germline phenotypes have been observed at the earlier age in *cep-1* mutants [67]. We identified that DIM supplementation slowed age-related decline of oocyte quality and induced the expression of CEP-1 in the aged germ line. It was suggested that the interaction within germ cells for the maintenance of a healthy gonad could be observed by whether the level of GCP was reduced by accumulating germ cells in apoptosis defects of *ced-3* mutants after ovulation [68]. Conceivably, both GCA and GCP induced by DIM supplementation required CEP-1 activity, and GCP level was decreased in *ced-3* mutants in this study, suggesting a possible link between GCA and GCP through CEP-1 activity. Additionally, CEP-1/p53 is involved in mitochondrial dysfunction and reproduction [69,70]. Although the *cep-1* single mutant did not affect the number of progenies, double mutants with either *isp-1* or *mev-1,* which show mitochondrial dysfunction, produced significantly reduced numbers of progenies [69]. Furthermore, in response to oxidative stress, CEP-1-mediated GCA maintains mitochondrial function in oocytes and the number of progenies [70]. Accordingly, these findings raise the possibility that CEP-1 could contribute to the reproductive capacity associated with mitochondrial function. Thus, we propose that DIM supplementation maintains germline and oocyte quality in reproductively aged worms by activating the CEP-1/p53 pathway.

**Figure 7:**
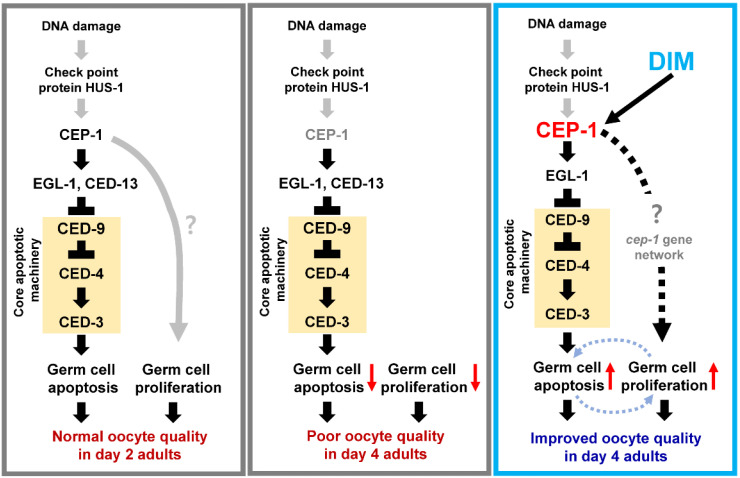
Working model: DIM supplementation maintains the level of GCA and GCP in the reproductively aged *Caenorhabditis elegans* germ line in the presence of CEP-1/p53 activity. GCA is mediated by CEP-1-EGL-1 pathway that inhibits CED-9 expression, which then activates core apoptotic machinery. Both GCA and GCP contribute to improving oocyte quality during reproductive aging.

Here, we show in vivo evidence that dietary supplementation with DIM maintains oocyte quality by improving mitochondrial function and resistance to oxidative stress, and germline quality in reproductively aged mothers at the organismal level as proposed in Figure 7. The improved oocytes show not only healthy mitochondria but also better chromosomal morphology. As a result, the embryos remained alive compared to those from control animals without DIM supplementation. The effects of DIM supplementation are still controversial, with both beneficial and harmful effects on fertility being reported. Therefore, it should be carefully noted that the beneficial effects on oocyte quality control in reproductively aged worms may not be conserved in other organisms. However, it is worthwhile verifying the role of DIM supplementation on reproductive capacity in aged animals at the molecular and organismal levels. Based on our findings, we propose that DIM is a beneficial natural antioxidant that enhances reproduction in *C. elegans*.

## 5. Conclusions

This study provides evidence of the beneficial effects of a natural antioxidant, DIM, on the quality of germ cells by maintaining GCA and GCP, and on oocyte quality by improving mitochondrial function and chromosomal integrity in a reproductively aged *C. elegans* model. These findings support the possibility that a natural dietary antioxidant can be a beneficial modulator to delay reproductive aging and thus reduce the risk of infertility in aging mothers.

## Data Availability

Data is contained within the article and Supplementary Material.

## Supplementary Materials

The following supporting information can be downloaded at: https://www.mdpi.com/article/10.3390/antiox11050950/s1, Figure S1: Structure of 3,3′-diindolylmethane; Figure S2: DIM supplementation suppresses the level of ROS in aged *prdx-2* mutants; Figure S3: The effect of DIM supplementation on reproductive capacity from the day 1 to day 6 post L4 stage; Figure S4: The mRNA levels of *egl-1* and *ced-13* in day 1 and day 4 adult worms with DIM supplementation; Figure S5: Three sets of western blot images; Figure S6: DIM supplementation did not induce the expression of SKN-1::GFP.
