# Supplementary figures and images for: 3,3′-Diindolylmethane Supplementation Maintains Oocyte Quality by Reducing Oxidative Stress and CEP-1/p53-Mediated Regulation of Germ Cells in a Reproductively Aged Caenorhabditis elegans Model
Source: Antioxidants (Basel). 2022 May 11;11(5):950. doi: 10.3390/antiox11050950 (PMC9137721; doi:10.3390/antiox11050950)

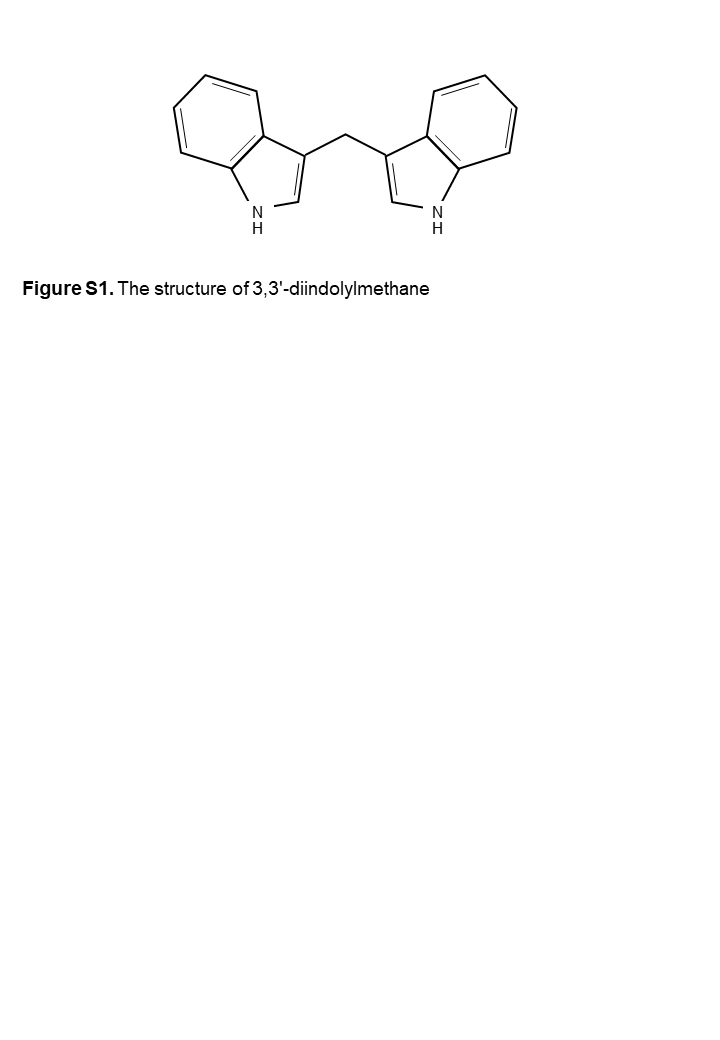

Supplement: Supplementary file 1 [file antioxidants-11-00950-s001.zip › Fig S1.TIF]

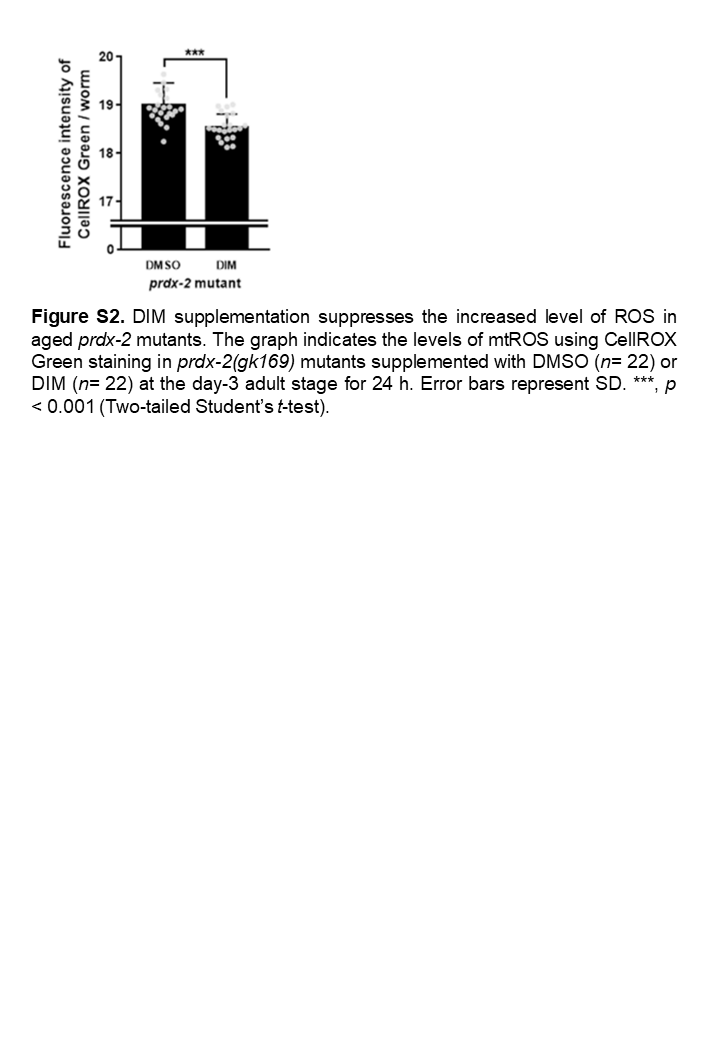

Supplement: Supplementary file 1 [file antioxidants-11-00950-s001.zip › Fig S2.TIF]

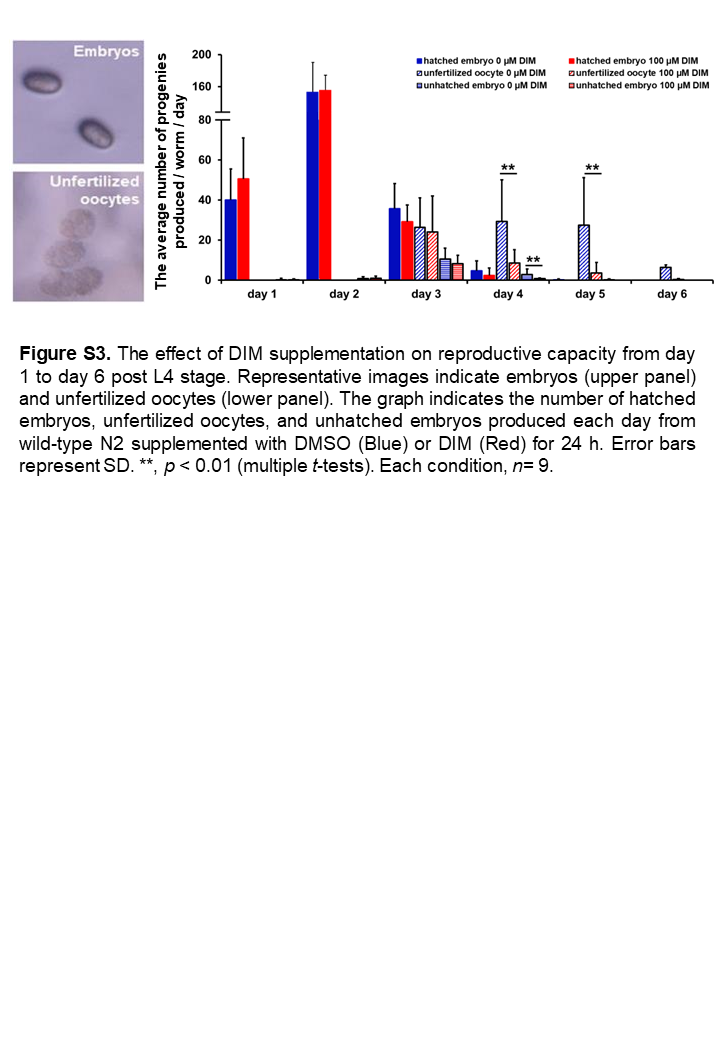

Supplement: Supplementary file 1 [file antioxidants-11-00950-s001.zip › Fig S3.TIF]

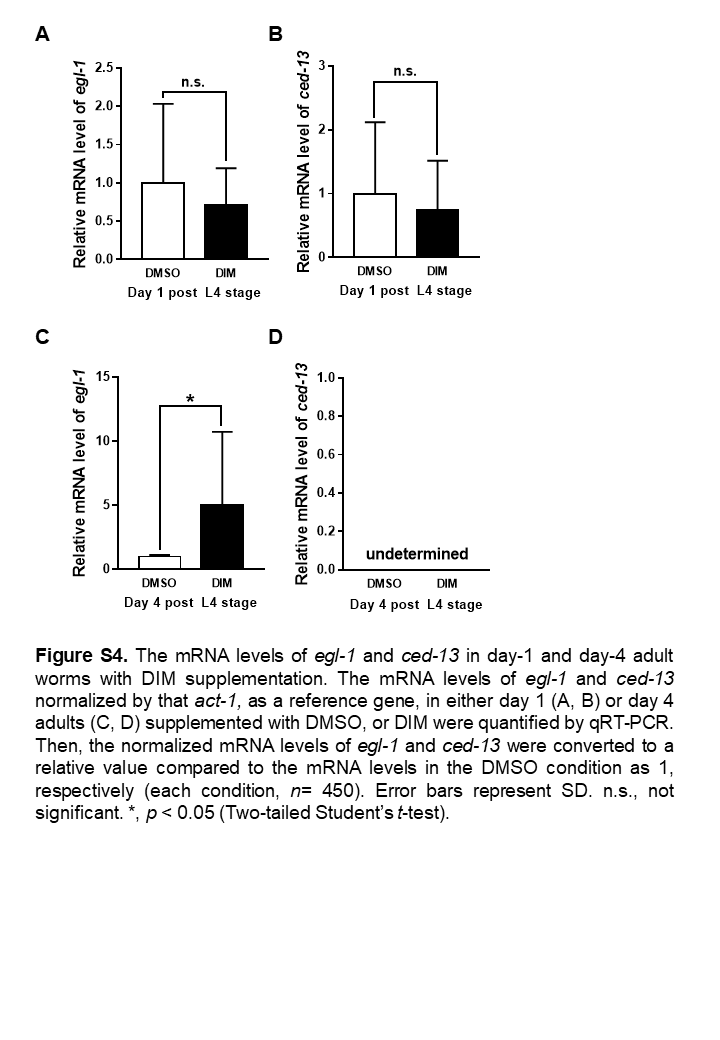

Supplement: Supplementary file 1 [file antioxidants-11-00950-s001.zip › Fig S4.TIF]

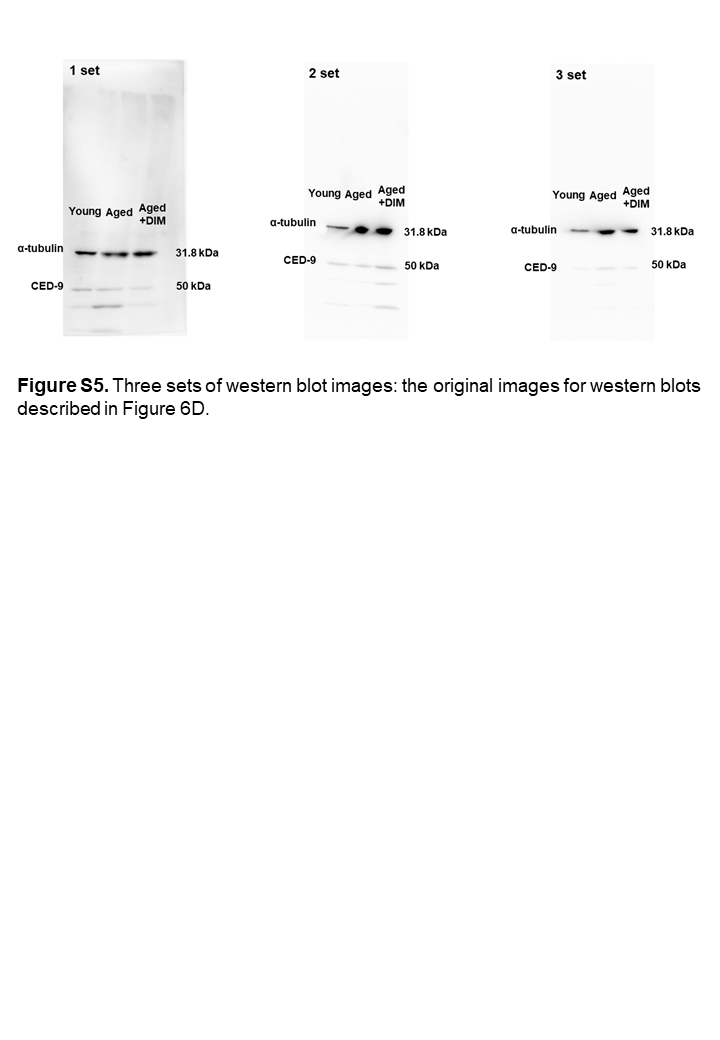

Supplement: Supplementary file 1 [file antioxidants-11-00950-s001.zip › Fig S5.TIF]

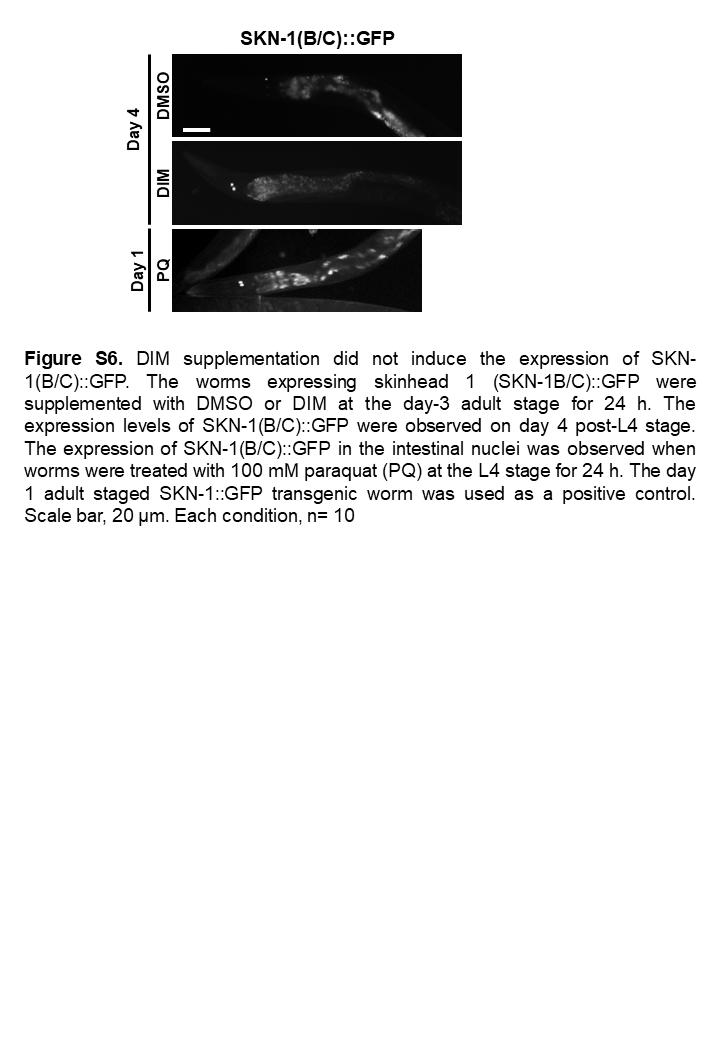

Supplement: Supplementary file 1 [file antioxidants-11-00950-s001.zip › Fig S6.TIF]
